# The Trimeric Autotransporter Adhesin YadA of *Yersinia enterocolitica* Serotype O:9 Binds Glycan Moieties

**DOI:** 10.3389/fmicb.2021.738818

**Published:** 2022-02-01

**Authors:** Ina Meuskens, Juan Leva-Bueno, Paul Millner, Monika Schütz, Sally A. Peyman, Dirk Linke

**Affiliations:** ^1^Section for Genetics and Evolutionary Biology, Department of Biosciences, University of Oslo, Oslo, Norway; ^2^Faculty of Biological Sciences, School of Biomedical Sciences, University of Leeds, Leeds, United Kingdom; ^3^Interfakultäres Institut für Mikrobiologie und Infektionsmedizin Tübingen (IMIT), Institut für Medizinische Mikrobiologie und Hygiene, Universität Tübingen, Tübingen, Germany; ^4^Molecular and Nanoscale Physics Group, Department of Physics and Astronomy, University of Leeds, Leeds, United Kingdom

**Keywords:** trimeric autotransporter adhesin, bacterial adhesion, virulence, extracellular matrix (ECM), adhesion, glycan

## Abstract

*Yersinia* adhesin A (YadA) is a key virulence factor of *Yersinia enterocolitica* and *Yersinia pseudotuberculosis*. YadA is a trimeric autotransporter adhesin, a class of adhesins that have been shown to enable many Gram-negative pathogens to adhere to/interact with the host extracellular matrix proteins such as collagen, vitronectin, and fibronectin. Here, we show for the first time that YadA of *Yersinia enterocolitica* serotype O:9 not only interacts with proteinaceous surface molecules but can also attach directly to glycan moieties. We show that YadA from *Y. enterocolitica* serotype O:9 does not interact with the vitronectin protein itself but exclusively with its N-linked glycans. We also show that YadA can target other glycan moieties as found in heparin, for example. So far, little is known about specific interactions between bacterial autotransporter adhesins and glycans. This could potentially lead to new antimicrobial treatment strategies, as well as diagnostic applications.

## Introduction

*Yersinia* adhesin A (YadA), a type Vc trimeric autotransporter adhesin of *Yersinia* spp. is crucial for virulence. YadA is encoded on a virulence plasmid, the pYV plasmid. Expression of the YadA gene is temperature controlled, and upon a temperature shift to 37°C, once the bacterium enters the host, the expression of YadA is initiated (Toivanen and Skurnik, 1992).

YadA is a surface-exposed adhesin that is anchored in the bacterial outer membrane *via* a trimeric β-barrel domain (Shahid et al., 2015). The passenger domain of YadA, a trimeric coiled-coil stalk, and an N-terminal β-roll head domain are channeled through the barrel in an unfolded state during the autotransport process (Chauhan et al., 2019). Upon autotransport, the passenger domain starts folding, building a rigid structure protruding toward the outside of the cell (Chauhan et al., 2019). Here, the head domain has been shown to be responsible for many of YadA’s adhesion properties (Leo et al., 2008; Mühlenkamp et al., 2015).

**FIGURE 1:**
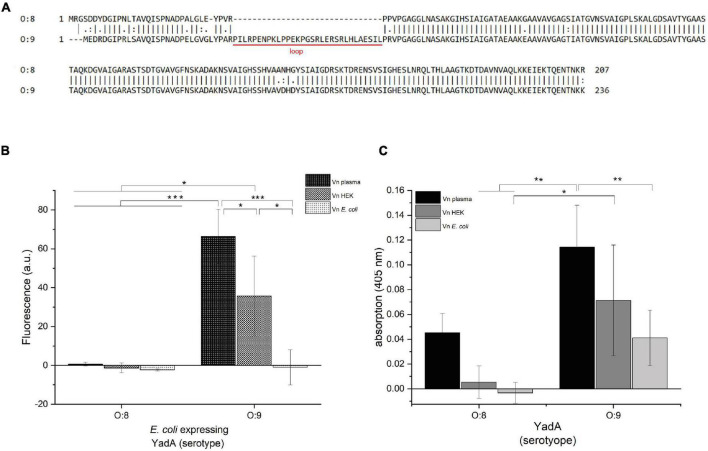
YadA_*O:*9_ binds to Vn_plasma_ and Vn_HEK_ but shows reduced binding to Vn_Ec_. **(A)** Alignment of the N-terminal head domains of the YadA head domain from *Y. enterocolitica* serotypes O:8 and O:9. The alignments include the head domain of YadA, the neck region, and the first 20 residues of the coiled-coil stalk domain. YadA_*O:*9_ has an insertion of 31 residues toward the N-terminus of the head domain. **(B)** Whole-cell binding assay to Vn using *E. coli* expressing either YadA_*O:*8_ or YadA_*O:*9_. **(C)** Vn binding assay with purified YadA_*O:*8_ and YadA_*O:*9_ head domains. Significance levels are indicated with *p < 0.05, **p < 0.01, ***p < 0.001.

While YadA is typically classified as an adhesin that aids in pathogen–host interactions *via* interactions with the extracellular matrix (ECM) (Tamm et al., 1993; Westerlund and Korhonen, 1993; Leo et al., 2008; Keller et al., 2015), YadA has also been shown to be involved in immune evasion (Tamm et al., 1993; Westerlund and Korhonen, 1993; Grosskinsky et al., 2007; Leo et al., 2008; Schindler et al., 2012; Keller et al., 2015). YadA-knockout mutants are avirulent (Pepe et al., 1995; Schütz et al., 2010). This is only partially due to the adhesion properties of YadA as *Yersinia* spp. have additional adhesins which can replace its function (Mallick et al., 2012; Chauhan et al., 2016). During an infection with *Yersinia enterocolitica*, YadA is involved in surface adhesion and has been shown to interact with a variety of proteinaceous ECM molecules such as collagen, fibronectin, and vitronectin (Vn) (Tertti et al., 1992; Schulze-Koops et al., 1993; Leo et al., 2008; Mühlenkamp et al., 2017). The interaction with ECM varies in strength and depends on environmental shear forces (Müller et al., 2011). While the YadA head domain is conserved among *Yersinia* species, some *Y. enterocolitica* strains and *Yersinia pseudotuberculosis* exhibit an additional stretch of approximately 31 residues at the N-terminus of each monomer of the YadA head domain (Figure 1A). This stretch has been shown to be responsible for Vn binding (Mühlenkamp et al., 2017).

Vn has been described as an incidental component of the ECM (Leavesley et al., 2013). The ECM is a matrix composed of a variety of proteins, such as collagen, fibronectin, and laminin, and also proteoglycans and glycosaminoglycans (GAGs) forming a hydrogel. This matrix surrounds cellular components of the cell surface and provides strength and elasticity (Frantz et al., 2010). Vn is an approximately 75 kDa glycoprotein involved in tissue repair. It is heavily glycosylated, exhibiting three N-linked glycans (N86, N169, and N242) (Hwang et al., 2014). Vn shows great flexibility, and its conformational state is greatly dependent on interaction partners such as heparin (Izumi et al., 1989; Stockmann et al., 1993). The ability of Vn to associate with GAGs like heparin and heparan sulfate, which are in turn part of the ECM, contributes to the function of Vn in tissue repair (Leavesley et al., 2013). The ECM and its components are an attractive binding target for *Y. enterocolitica* and *Y. pseudotuberculosis* as surface adhesion is crucial for subsequent tissue invasion (Pepe et al., 1995).

Here, we report that YadA from *Y. enterocolitica* strain E40, serotype O:9 (YadA_*O:*9_) interacts with Vn *via* its glycosylations. We furthermore show that YadA_*O:*9_ can directly interact with heparin. Up until now, an interaction with glycan moieties like the glycosylations of ECM proteins or GAGs has not been described for YadA.

## Materials and Methods

### Plasmids and Constructs

Plasmids and constructs used in this study are listed in Table 1, and sequences can be found in the supplements (Table 1). Constructs made in this study were cloned using the Gibson assembly (Gibson et al., 2009).

**TABLE 1:** Constructs used in this study.

Construct	Resistance	Source strain	Source
pASK-IBA4C_YadA_*O:8*_	Chloramphenicol	*Y. enterocolitica* O:8 WA-314	Mühlenkamp et al., 2017
pASK-IBA4C_YadA_*O:9*_	Chloramphenicol	*Y. enterocolitica* O:9 E40	Mühlenkamp et al., 2017
pASK-IBA3_YadA_*O:9*__head domain	Ampicillin	*Y. enterocolitica* O:8 WA-314	This study (supplement)
pASK-IBA3_YadA_*O:9*__head domain	Ampicillin	*Y. enterocolitica* O:9 E40	This study (supplement)

### Bacterial Strains and Growth Conditions

Bacteria were cultivated in Lysogeny broth (LB, Miller formulation). For whole-cell assays with fluorescence detection, *Escherichia coli* Top10 *glmS*:sfGFP (AS75) was used and grown in the presence of arabinose (Saragliadis and Linke, 2019). For protein purification, genes encoding the proteins of interest were expressed in *E. coli* BL21 (DE3) Gold. Generally, bacteria were grown at 37°C to the desired OD_600_. During overexpression, the temperature was shifted to 23°C after induction.

### YadA Head Domain Purification

pASK-IBA3_YadA_*O:*8/O:9_ was transformed into *E. coli* BL21 (DE3) Gold and grown on ampicillin plates. A single colony was inoculated into 20 ml of LB medium supplemented with 100 μg/ml ampicillin and grown at 37°C overnight (o/n). The following day, a 2 L subculture was prepared and grown in a home-built fermentation system (a system where air is bubbling through bottles of growth medium that stand in a temperature-controlled water bath) until an OD_600_ of 0.5–0.7 was reached. The temperature was shifted to 23°C, and expression was induced with 0.2 μg/ml anhydrotetracycline (AHTC). Protein expression was allowed for 16 h. The culture was harvested by centrifugation at 4,000 × *g*. Afterward, the pellet was resuspended in Tris-buffered saline (TBS) buffer (20 mM Tris pH 7.5, 300 mM NaCl, 20 mM imidazole) with 8 μg/ml lysozyme and a pinch of DNAse. The suspension was subjected to cell lysis using a French press after addition of a HALT protease inhibitor mix (1:500, Thermo Fisher Scientific; 1861278). The lysate was centrifuged for 1 h at 69,600 × *g*, and the supernatant was then filtered through a 0.2 μm filter and subjected to Ni-NTA affinity chromatography (Cytiva, 17531901). As YadA with C-terminal His_6_-tag elutes at high imidazole concentrations (160–500 mM), the protein was pure enough for binding experiments after Ni-affinity chromatography. The protein was subjected to dialysis against TBS buffer (20 mM Tris pH 7.5, 150 mM NaCl).

### Vitronectin Binding Experiments With Whole Bacteria

*E. coli* AS75 with pASK-Iba4C_YadA_*O:*8_ or pASK-Iba4C_YadA_*O:*9_ was grown o/n in LB medium supplemented with 0.02% (w/v) arabinose and 100 μg/ml ampicillin. The next day, the cultures were diluted 1:100 in 20 ml LB medium supplemented as before and grown at 37°C to an OD_600_ of 0.5. YadA expression was then induced by the addition of AHTC to a final concentration of 0.2 μg/ml and grown for another 3 h at 37°C. YadA expression was checked for by visual inspection for auto-aggregation (Trunk et al., 2018). In the meantime, clear flat-bottom 96-well plates were coated with 100 μl of a 10 μg/ml Vn solution, from either plasma (Gibco, PHE0011), recombinantly expressed in HEK cell cultures (Merck/Millipore, SRP3186), or *E. coli* (Thermo Fisher Scientific, A14700), by incubation for 1 h at room temperature (RT). The Vn solution was discarded from the plates, and the wells were washed three times with TBS (20 mM Tris pH 7.5, 150 mM NaCl). Afterward, the wells were blocked using 3% bovine serum albumin (BSA) in TBS. The bacteria were then harvested, washed twice with TBS, and resuspended in TBS with 0.1% BSA to achieve an OD_600_ of 0.2. One hundred microliters of the bacterial suspension was added per well and incubated for 1 h at RT. After that, the wells were washed three times using TBS. Lastly, the wells were filled with 100 μl TBS buffer, and fluorescence was measured using an excitation wavelength of 488 nm and recording the emission at 533 nm (BioTek Synergy H). For experiments with deglycosylated Vn, the experiment was performed the same way, but deglycosylated Vn (see section “Deglycosylation of Vitronectin”) was used for coating.

### Vitronectin Binding Experiments With Purified YadA Head Domains

A clear 96-well plate was coated with Vn and blocked as described before (section “Vitronectin Binding Experiments With Whole Bacteria”). Then 100 μl of a 10 μg/ml YadA solution in 0.1% (w/v) BSA in TBS (20 mM Tris pH 7.5, 150 mM NaCl) was added to the wells and incubated for 1 h at RT. After the wells were washed twice with 0.1% BSA in TBS and once with TBS with Tween-20 (TBS-T), Ni-horseradish peroxidase (HRP) conjugates were used for the detection of bound, His_6_-tagged YadA. One hundred microliters of a Ni-HRP conjugate solution at a final concentration of 5 μg/ml (Thermo Fisher Scientific, 15165) was incubated for 1 h at RT in 3% BSA in TBS per well. This was discarded, and the wells were washed three times with TBS-T and once with TBS. Binding was detected using 150 μl of a 1 mg/ml ABTS solution in ABTS buffer (2.43 ml of 100 mM citric acid, 2.57 ml of 200 mM Na_2_HPO_4_, 5.0 ml H_2_O, 10 μl H_2_O_2_) (Thermo Fisher Scientific, 34026; VWR, ICNA0219502305). The color development was stopped by adding 100 μl of 1% (w/v) SDS after incubation at RT, and the absorption was measured at 405 nm in a BioTek Synergy H plate reader.

### Deglycosylation of Vitronectin

For deglycosylation of Vn, 20 μg of Vn from the respective sources (in water) was mixed with 2 μg of the glycopeptidase PNGase F (500 U) (Promega, V4831) and incubated at 37°C for 19 h. The non-deglycosylated control samples of Cn were incubated at 37°C for 19 h, omitting the PNGase F. For PNGase F control samples, 2 μl of PNGase F was added to water and incubated as described before. Successful deglycosylation was checked for on a SDS-PAGE gel with subsequent silver staining (Nesterenko et al., 1994).

### Heparin Inhibition Assay Using Microscopy

Glass coverslips were coated with 50 μl Vn (10 μg/ml) at 4°C o/n. An o/n culture of *E. coli* AS75 harboring pASK-IBA4C_YadA_*O:*8/O:9_ was inoculated into LB supplemented with 20 μg/ml chloramphenicol and 0.02% w/v arabinose. The next day, the culture was diluted 1:100 in the same broth, and the culture was grown to OD_600_ of 0.5 followed by induction with 0.2 μg/ml AHTC and *yadA* expression for 3 h at 37°C. In the meantime, Vn-coated coverslips were incubated with TBS or 100 μM heparin-disaccharide I-S (Merck, H9267-1MG) for 1 h at RT where applicable. After that, all coverslips were blocked with 3% (w/v) BSA in TBS for 1 h at RT. One hundred microliters of 5 × 10^8^ bacteria in suspension were centrifuged at 4,000 × g for 5 min and resuspended in either TBS (20 mM Tris pH 7.5, 150 mM NaCl) or 100 μM heparin-disaccharide in TBS and incubated for 1 h at RT. After that, the bacteria were centrifuged down again and washed three times in 100 μl TBS. Finally, the bacteria were resuspended in 1 ml 3% BSA in TBS. Three hundred microliters of the bacteria was added to the coverslips and incubated for 30 min at RT. The supernatant was discarded, and the coverslips were washed three times with TBS and fixed with 500 μl of 4% (w/v) paraformaldehyde in TBS for 20 min at RT. Finally, the coverslips were mounted in 5 μl ProLong Glass Antifade Mountant (Invitrogen, P36980) and dried o/n. Microscopy was performed using a fluorescent microscope (Zeiss Axioplan 2) and a 100× oil immersion objective. For quantification, images were converted into binary files, and the area of the particles was calculated using Fiji (Supplementary Figure 3). Mean areas were plotted including the standard error of the mean.

### Disaggregation Experiments Using Microscopy

An o/n culture of *E. coli* AS75 harboring pASK-IBA4C_YadA_*O:*8/O:9_ was inoculated into LB supplemented with 100 μg/ml ampicillin and 0.02% (w/v) arabinose. The next day, the culture was diluted 1:100 in the same broth and grown to OD_600_ of 0.5 followed by induction with 0.2 μg/ml AHTC and *yadA* expression for 3 h at 37°C. The culture was diluted to an OD_600_ of 1.0, and 50 μl was centrifuged down at 4,000 × *g* for 5 min. The pellets were then resuspended in 50 μl TBS or TBS supplemented with 100 μM heparin-disaccharide (Merck, H9267-1MG). This was incubated at 37°C in a shaking incubator for 30 min. Five microliters of each solution was wet-mounted onto microscope slides, and the edges were sealed using a CoverGrip coverslip sealant (Biotium, 23005). Microscopy was performed using a fluorescence microscope (Zeiss Axioplan 2) and a ×100 oil immersion objective. For quantification, images were converted into binary files, and the particle sizes were calculated using Fiji (Supplementary Figure 4). The area of each individual particle was plotted in a column scatter plot.

### Dot Blots for Heparin Binding to YadA Head Domains

Nitrocellulose membranes were cut and transferred into a six-well plate. Three 2 μl drops of a 700 μg/ml purified YadA_*O:*8_ or YadA_*O:*9_ solution were applied onto the membrane and air-dried. Then, the membrane was blocked with 5% BSA in TBS-T (20 mM Tris pH 7.5, 150 mM NaCl, 0.2% Tween-20) for 1 h at RT. Five hundred microliters of a 100 μM biotinylated heparin (Merck, B9806-10MG) solution in TBS-T was incubated on the membrane for 1 h at RT. The membrane was washed three times with TBS-T and afterward incubated with 500 μl of 1:10,000 diluted Strep-Tactin–HRP conjugate (IBA Lifesciences, 2-1502-001) in 5% BSA in TBS-T for 30 min at RT. After the membrane was washed three times with TBS-T and once with TBS (20 mM Tris pH 7.5, 150 mM NaCl), a 500 μl ECL reagent (Thermo Fisher Scientific, 320106) was added, and the membrane was immediately imaged using a Kodak Image Station 4000R.

### Heparin Binding Assay Using Bacteria

An o/n culture of *E. coli* AS75 pASK-IBA4C_YadA_*O:*8/O:9_ was grown in the presence of 0.2% (w/v) arabinose and 100 μg/ml ampicillin. This culture was diluted 1:100 the next morning and grown to an OD_600_ of 0.5. YadA full-length expression was induced by addition of 0.2 μg/ml AHTC. Expression was allowed for 3 h at 37°C. Uninduced bacteria were used as a control. The bacteria were diluted to an OD_600_ of 0.2, spun down, and resuspended in phosphate-buffered saline (PBS). One hundred microliters of that bacterial solution was pipetted into 96-well plates and centrifuged at 4,000 × *g*. After that, 100 μl of a 10 μg/ml biotinylated heparin (Merck, B9806-10MG) solution in 3% (w/v) BSA in TBS was added and incubated at RT for 0.5 h. The plate was washed three times with TBS. The plate was centrifuged as before after every wash before discarding the washing buffer. Strep-Tactin–HRP conjugates (IBA Lifesciences, 2-1502-001) at 1:1,000 in 3% (w/v) BSA in TBS were added and incubated for 30 min at RT. The plate was washed as described before. The ABTS solution was prepared, and color development was stopped as described before. Wells that did not contain any bacteria were used as background controls. Absorbance at 405 nm was measured in a plate reader (BioTek Synergy H).

### Binding Assay Using Purified YadA Head Domains

One hundred microliters of 10 μg/ml YadA in TBS was coated into a 96-well plate by incubation at RT for 1 h. The plate was washed three times with 200 μl TBS (20 mM Tris pH 7.5, 150 mM NaCl) and blocked using 200 μl of 3% BSA in TBS. Afterward, 100 μl of biotinylated heparin dilution (0–6.75 μg/ml) in TBS was added to the wells and incubated for 1 h at RT. The wells were washed three times with TBS as described above and blocked with 3% BSA in TBS for 1 h at RT. Strep-Tactin–HRP (IBA Lifesciences, 2-1502-001) at 1:1,000 was added in 3% BSA in TBS and incubated for 1 h at RT. The wells were washed again as described earlier, and an ABTS solution was used for detection as described before. After color development, the reaction was stopped by adding 100 μl of a 1% SDS solution. Absorbance at 405 nm was measured in a plate reader (BioTek Synergy H).

### Binding Assay Using Immobilized Heparin on Impedimetric Nanobiosensors

#### Biosensor Fabrication

Gold screen-printed electrodes (BVT-AC1.W1.RS.Dw2) from BVT Technologies were employed for biosensor fabrication. The electrodes were pre-treated by washing with 97% v/v ethanol for 30 min, rinsed with deionized water, and dried with N_2_. Twenty-five microliters of 2.5 mM octopamine in 10 mM phosphate buffer pH 7.2 was spread across the working electrode and electro-polymerized for two cycles at a scan rate of 100 mV/s from +0.0 to +1.6 V. The electrodes were rinsed with 10 mM PBS and dried with Ar. The electrodes were functionalized by binding of biotinylated NeutrAvidin (Ahmed et al., 2013). After that, 10 μl of a 1 mg/ml biotinylated heparin was coated onto the surface for 1 h at RT. The surface was washed with 10 mM PBS and dried with Ar.

#### Electrochemical Impedance Spectroscopy Measurements and Data Treatment

For binding measurements, 10 μl of *E. coli* AS75 expressing either YadA_*O:*8_ or YadA_*O:*9_ full length at OD_600_ of 2, 0.2, and 0.02 was applied to the working electrodes and incubated for 30 min at RT. Blanks were acquired by measuring 10 mM phosphate buffer, omitting the bacteria. Electrical impedance measurements were carried out in a three-cell system of a PalmSens4 potentiostat, galvanostat, and frequency response analyzer (PalmSens BV, Netherland), adding 10 mM [Fe(CN)6]^3–/4–^ in 10 mM PBS pH 7.2 onto the electrodes. EIS measurements were recorded at 0 V over a frequency range of 5–0.1 Hz, with a modulation voltage of + 10 mV. Measurements corresponding to finite Warburg impedances were excluded from the Nyquist plots (Nguyen and Breitkopf, 2018). PSTrace (5.8) was used to record the EIS measurements. Metrohm Autolab Nova 2.1.4. was used to fit the Nyquist plots into Randles’ equivalent circuits. From the fitting, the charge-transfer resistance (Rct) was obtained. The biosensor was assessed before and after analyte addition. Changes in Rct (%) were obtained to analytically assess bacterial binding with Equation 1:


(1) ChangeinRct(%)=(Rct-analyteRct)zero/Rct⋅zero100

All experiments were replicated *n* ≥ 6. Layer-by-layer construction can be found in Supplementary Figure 2.

### Statistical Analysis

For binding data analysis, data are shown as means ± SD and were analyzed using a one-way ANOVA including Tukey’s test. For data plotting and statistical analysis, OriginPro and “R” were used. For microscopy analysis, mean particle areas ± SEM were plotted. As the particles sizes were not normally distributed, non-parametric testing including a Kruskal–Wallis test and subsequent Wilcox testing were applied to test for significance. Significance levels are indicated in the graphs with *p* < 0.05 (*), *p* < 0.01 (^**^), or *p* < 0.001 (^***^).

## Results

### Head Domain of YadA From *Y. enterocolitica* Serotype O:9 Binds to Vitronectin Isolated From Plasma and HEK Cells but Not to Vitronectin Produced in *E. coli*

We first aimed to describe the molecular details of the interaction between the YadA head domain of *Y. enterocolitica* serotype O:9 (YadA_*O:*9_) and Vn. YadA_*O:*9_ harbors an additional, N-terminal, 31-residue stretch (Figure 1A) that has been described to interact with Vn (Mühlenkamp et al., 2017). We started out replicating the experiments done by Mühlenkamp et al. (2017). For these enzyme-linked immunosorbent assay (ELISA)-like binding experiments, Vn from different sources was used. Vn purified from plasma (Vn_plasma_), Vn expressed in HEK cell culture (Vn_HEK_), and Vn recombinantly expressed in *E. coli* (Vn_Ec_) were tested for their capacity to be bound by *E. coli* expressing either full-length YadA_*O:*8_ or YadA_*O:*9_ (Figure 1B). Additionally, Vn binding by purified YadA head domains was tested (Figure 1C). We reasoned that, if YadA indeed bound to a conserved sequence within Vn, the binding should happen irrespective of the origin of Vn and only with YadA_*O:*9_. Indeed, the whole-cell assays show that only YadA_*O:*9_-expressing bacteria bound to Vn_plasma_ and Vn_HEK_ (Figure 1B). Binding between Vn_Ec_ and YadA_*O:*9_ could not be observed. Bacteria expressing YadA_*O:*8_ did not bind to either Vn variant (Figure 1B). These findings were corroborated by assays using purified YadA head domains. While we observed some binding of purified YadA_*O:*8_ to Vn_plasma_, no binding to Vn_HEK_ and Vn_*Ec*_ was observed (Figure 1C). The weak binding of purified YadA_*O:*8_ can be explained by Vn_plasma_ being contaminated with other proteins (Supplementary Figure 1). YadA_*O:*9_ on the other hand showed clear binding to all Vn variants, with reduced binding to Vn_Ec_. Based on these findings, we sought to investigate the difference between YadA_*O:*9_ binding to Vn_plasma/HEK_ and binding to Vn_Ec_.

### The Head Domain of YadA From *Y. enterocolitica* Serotype O:9 Only Binds Glycosylated Vitronectin

**FIGURE 2:**
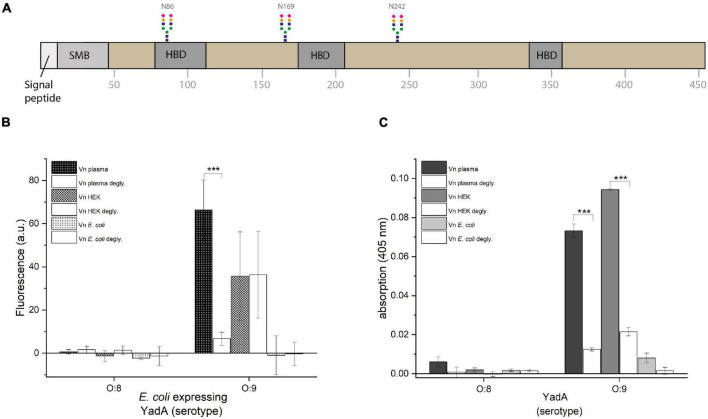
YadA_*O:*9_ binding to deglycosylated Vn is reduced. **(A)** Schematic representation of full-length Vn. Vn has three N-linked glycosylations in positions N86, N169, and N242. **(B)** Whole-cell binding assay to glycosylated and deglycosylated Vn. **(C)** Binding assay using purified YadA head domains to glycosylated and deglycosylated Vn. Significance levels are indicated with *p < 0.05, **p < 0.01, ***p < 0.001.

Due to the observation that YadA_*O:*9_ binds Vn_plasma_ and Vn_*HEK*_ but shows at least reduced binding to Vn_Ec_, we wanted to investigate whether YadA_*O:*9_ actually binds a stretch within Vn or whether it either recognized a folded binding site or the glycosylations of Vn. Vn is heavily glycosylated with at least three N-linked glycans at residues N86, N169, and N242 (Figure 2A). As eukaryotic proteins recombinantly expressed in *E. coli* are usually not glycosylated, we first tested the latter hypothesis. We used PNGase F, a glycopeptidase that selectively removes glycans directly at the N-linkage by cleaving the glycosidic bond between asparagine and the core GlcNAc. With the deglycosylated Vn, the binding assays were repeated to see whether binding could be abrogated by removal of the N-linked glycosylations. In Figure 2B, the fluorescence-based whole-cell assay using *E. coli* AS75 expressing either full-length YadA_*O:*8_ or full-length YadA_*O:*9_ is shown. No binding was observed with cells expressing YadA_*O:*8_, which fits the hypothesis, as the postulated Vn binding stretch is not present in YadA from *Y. enterocolitica* serotype O:8. In the case of binding of bacteria expressing full-length YadA_*O:*9_, a clear difference in binding to Vn_plasma_ was observed between the glycosylated Vn_plasma_ and deglycosylated Vn_plasma_ (Figure 2B). For Vn_HEK_, no change in binding of YadA_*O:*9_-expressing *E. coli* AS75 before and after glycosylation was observed. We can at this point not say as to why no change was observed for bacterial binding of Vn_HEK_ compared to deglycosylated Vn_HEK_. Vn_Ec_ was bound in neither the glycosylated nor the deglycosylated state. While this supported our hypothesis that the glycan residues of Vn might be involved in the YadA_*O:*9_–Vn interaction rather than the proteinaceous part of Vn, we also repeated the binding assay using purified YadA head domains from both serotypes of *Y. enterocolitica* (Figure 2C). While, as expected, YadA_*O:*8_ did not bind to Vn-coated plates, neither to the untreated nor to the deglycosylated version, YadA_*O:*9_ bound to both untreated Vn_plasma_ and untreated Vn_HEK_ (Figure 2C). Untreated Vn_Ec_ was not bound as already shown in Figure 1C. After deglycosylation with PNGase F, neither Vn_plasma_ nor Vn_HEK_ was bound by YadA_*O:*9_ anymore, further supporting our hypothesis of YadA_*O:*9_ interacting with the N-linked glycans.

### Binding of YadA_*O:*9_ to Heparin Abrogates the YadA_*O:*9_–Vitronectin Interaction

**FIGURE 3:**
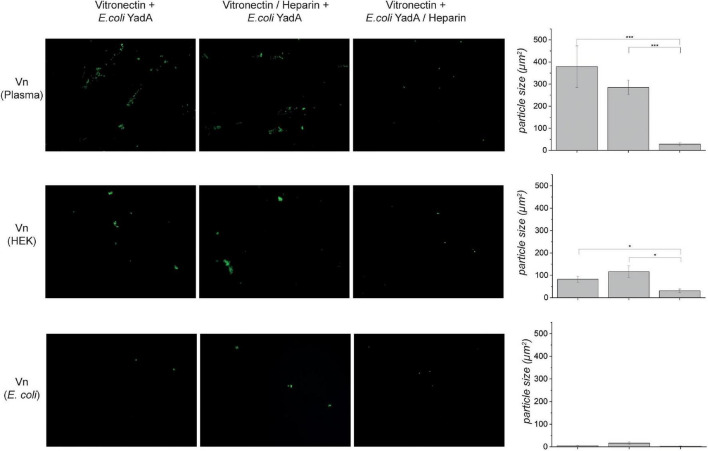
Binding of *E. coli* expressing YadA_*O:*9_ is reduced when the bacteria are preincubated with heparin-disaccharide. Vn from different sources was coated onto coverslips, and binding of fluorescence *E. coli* expressing YadA_*O:*9_ was measured (left column). Images in the middle column show micrographs of Vn-coated coverslips that were preincubated with 100 μM heparin-disaccharide. Afterward, binding of fluorescent *E. coli* expressing YadA_*O:*9_ was monitored. When Vn was coated and binding of fluorescent *E. coli* expressing YadA_*O:*9_ that were preincubated with heparin was (column 3) reduced, adhesion was measured for both Vn_plasma_ and Vn_HEK_. *E. coli* expressing YadA_*O:*9_ did only weakly bind to Vn_Ec_ as already observed in previous experiments. In the last column, the mean area of particles (μm^2^) ± SEM was calculated and plotted as bar graphs. Significance levels are indicated with *p < 0.05, **p < 0.01, ***p < 0.001.

Heparin was described to abrogate the interaction between Vn and YadA_*O:*9_ (Mühlenkamp et al., 2017). We next wanted to investigate whether the potential YadA_*O:*9_ glycan interaction might be the cause for this observation. It was hypothesized before that heparin blocks the YadA binding site on Vn. As in the globular state, the heparin binding site in Vn is mostly hidden inside the core of the protein; this seemed unlikely to be the reason for YadA_*O:*9_ binding inhibition (Hayashi et al., 1985; Izumi et al., 1989; Zhuang et al., 1996; Leavesley et al., 2013). Coverslips were coated with untreated Vn_plasma_, Vn_HEK_, or Vn_Ec_. YadA_*O:*9_ (full length)-expressing, fluorescent bacteria were checked for binding (Figure 3, left column). To check for the influence of heparin on this interaction, we also prepared samples where we either preincubated Vn with heparin (Figure 3, middle column) or preincubated YadA_*O:*9_-expressing bacteria with heparin (Figure 3, right column). In the fluorescence microscopy adhesion assay, we observed only minimal adhesion of bacteria to Vn_Ec_ (Figure 3, bottom row). When coverslips had been coated with Vn_plasma_ or Vn_HEK_, adhesion was observed only in the absence of heparin. In cases where Vn was preincubated with heparin, bacteria expressing YadA_*O:*9_ adhered to Vn to a comparable level as in the untreated samples (Figure 3, left and middle columns). When YadA_*O:*9_-expressing bacteria were preincubated with heparin, reduced binding to untreated Vn_plasma_ and Vn_HEK_ was observed (Figure 3, right column). Quantifications of the area of the particles reflect the tendencies seen in the experiment, where preincubation of the bacteria expressing YadA_*O:*9_ with heparin seems to reduce binding to Vn whereas preincubation of Vn with heparin did not change the adhesion of YadA_*O:*9_-expressing bacteria. This observation further strengthened our hypothesis that the YadA Vn-binding loop aids in adhesion of YadA_*O:*9_ to glycan moieties.

### The Head Domain of YadA From *Y. enterocolitica* Serotype O:9 Prefers Heparin Binding Over Autoaggregation

**FIGURE 4:**
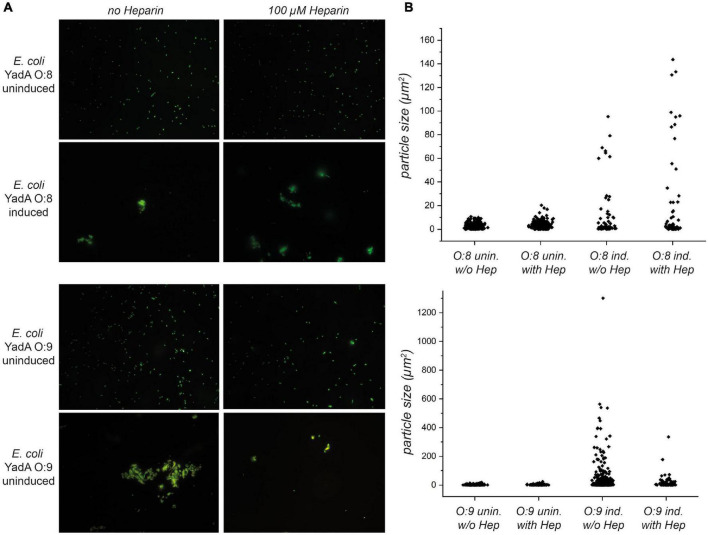
Autoaggregation of *E. coli* expressing YadA_*O:*9_ in the presence of 100 μM heparin-disaccharide is reduced. **(A)** In the upper row, uninduced bacteria expressing YadA_*O:*8_ in the presence and absence of heparin-disaccharide are shown. Uninduced bacteria (no YadA_*O:*8_ expressed) were used as a negative control for aggregation. After induction, *E. coli* expressing YadA_*O:*8_ show autoaggregation (row 2). In rows 3 and 4, the autoaggregation behavior of *E. coli* expressing YadA_*O:*9_ was tested in the presence and absence of 100 μM heparin-disaccharide. Uninduced *E. coli* expressing YadA_*O:*9_ are shown in row 3, and induced samples are shown in row 4. **(B)** Quantifications of the area of the particles are shown in the bar scatter plots for *E. coli* expressing YadA_*O:*8_ samples in the upper graph and *E. coli* expressing YadA_*O:*9_ samples in the lower graph.

YadA, as an adhesin, is involved in autoaggregation, which has been described as an important mechanism for immune evasion during infection as well as for biofilm formation (Trunk et al., 2018). We have observed earlier that the interaction with other adhesin targets, such as ECM molecules, interferes with autoaggregation (manuscript in preparation). We thus wanted to investigate what effect heparin might have on autoaggregation mediated by YadA_*O:*9_. We expressed YadA_*O:*8_ or YadA_*O:*9_ full length in fluorescent *E. coli* AS75 and allowed for autoaggregation of these samples. Uninduced samples served as a control. Half of the samples were then preincubated with heparin-disaccharide. The uninduced samples did not show any autoaggregation behavior, either in the presence or in the absence of heparin (Figure 4, rows 1 and 3). The induced YadA_*O:*8_ samples autoaggregated to similar degrees both in the absence and in the presence of heparin (Figure 4, row 2). Fluorescent bacteria expressing YadA_*O:*9_ showed autoaggregation in the absence of heparin but reduced autoaggregation in the presence of heparin (Figure 4, lower row). This indicates that, indeed, heparin binding of YadA_*O:*9_ dissolves the autoaggregation tendencies caused by surface expression of YadA_*O:*9_. The dispersion of particles sizes (μm^2^) is shown in Figure 4B. The scatter plots reflect the difference in aggregate (particle) sizes. A reduction in area between aggregates of *E. coli* expressing YadA_*O:*9_ with and without addition of heparin-disaccharide can be seen shifting from large aggregates to smaller aggregates or fully disaggregated samples.

### YadA Directly Binds Heparin

**FIGURE 5:**
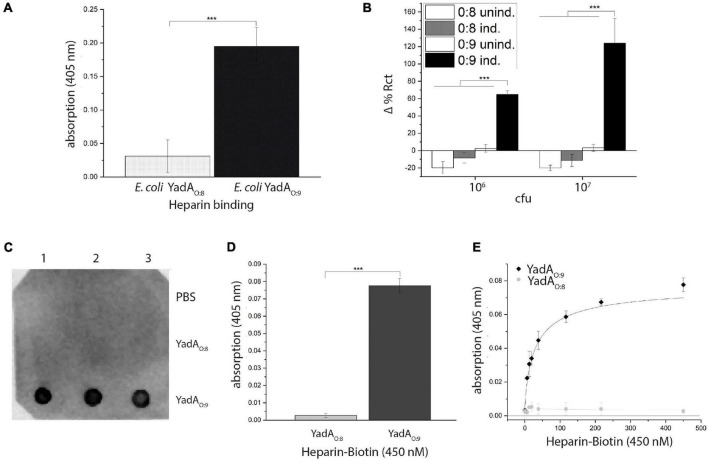
YadA_*O:*9_ directly binds to heparin. **(A)** Whole-cell binding assay of heparin to *E. coli* expressing YadA_*O:*8_ or YadA_*O:*9_ shows binding of YadA_*O:*9_-expressing bacterial binding to heparin. **(B)** Impedimetric biosensor experiment showing the adhesion of bacteria expressing YadA_*O:*8_ or YadA_*O:*9_ to surface-coated heparin. **(C)** Dot blot using immobilized YadA_*O:*8_ or YadA_*O:*9_ head domains shows direct binding to biotinylated heparin. **(D)** ELISA-like binding assay using immobilized YadA head domains to capture biotinylated heparin. **(E)** ELISA-like binding assay showing the interaction between immobilized, purified YadA head domains and biotinylated heparin in a concentration-dependent manner. Significance levels are indicated with *p < 0.05, **p < 0.01, ***p < 0.001.

To test for a direct interaction between YadA_*O:*9_ and heparin, we used *E. coli* AS75 cells expressing either YadA_*O:*8_ or YadA_*O:*9_. The bacteria were immobilized in a 96-well plate to capture biotinylated heparin. Bound biotinylated heparin was detected using Strep-Tactin–HRP. While no binding of biotinylated heparin to YadA_*O:*8_-expressing bacteria was observed, bacteria expressing YadA_*O:*9_ clearly showed heparin binding (Figure 5A). To support these results, we used electrochemical impedance measurements to measure bacterial binding to a heparin-coated surface. Biotinylated heparin was coated onto a biosensor using matrix-embedded NeutrAvidin (Ahmed et al., 2013). The change in impedance was then measured upon binding of *E. coli* AS75 expressing either YadA_*O:*8_ or YadA_*O:*9_ (Figure 5B). Please note that negative binding values are due to stronger adhesion of *E. coli* expressing YadA_*O:*8/O:9_ to uncoated electrodes that were used as a background and subtracted. While, for uninduced *E. coli* AS75 and *E. coli* expressing YadA_*O:*8_, no change in impedance was observed, we could clearly measure binding of *E. coli* expressing YadA_*O:*9_ by a significant change of impedance (Figure 5B). We then aimed to test for binding of heparin to purified YadA head domains. A dot blot using immobilized YadA_*O:*8_ and YadA_*O:*9_ head domains to detect binding of biotinylated heparin was performed. While no heparin binding was observed for either the buffer control or the YadA_*O:*8_ head domain, a signal could be observed for the binding of biotinylated heparin to the immobilized YadA_*O:*9_ head domain (Figure 5C). To quantify the binding, we immobilized the head domains of YadA_*O:*8_ and YadA_*O:*9_ in a 96-well plate and tested for binding at various concentrations. We observed that at 450 μg/ml of heparin, binding between YadA_*O:*8_ or YadA_*O:*9_ head domains and heparin is significantly different (Figures 5D,E). Repeating the assay with a dilution series of biotinylated heparin allowed us to investigate the concentration dependency of the binding. Using a fifth-party logistics fit, we estimate the (apparent) K_*D*_ to be approximately 30 nM. Furthermore, this experiment allows for an estimation of the binding ratio between YadA_*O:*9_ and biotinylated heparin. The binding ratio is estimated to be 1:1 (YadA_*O:*9_ monomer to biotinylated heparin). We can at this point not claim an accurate K_*D*_ or binding ratio as heparin varies in length but averages at 15 kDa (Shriver et al., 2012).

## Discussion

With this work, we present evidence that a 31-residue loop insertion specifically found in YadA_*O:*9_ is responsible for the interaction between YadA_*O:*9_ and glycan moieties. All experiments presented in this work were done using YadA from *Y. enterocolitica* strains WA-314 (serotype O:8) or E40 (serotype O:9). While Vn binding results published previously indicate that all *Y. enterocolitica* strains of serotype O:9 harbor this N-terminal 31-residue loop (Mühlenkamp et al., 2017), we cannot be sure that the presence or absence of this loop correlates with the serotypes in all cases. To our knowledge, sequence variations of YadA have never been reported to contribute directly to serotyping.

This loop region aids in the interaction between YadA_*O:*9_ and the glycosylated host protein Vn as well as heparin, which, like Vn, is part of the ECM. We show that this interaction is not specific for one type of glycan residue but rather for a variety of glycans. This is supported by the observation that YadA_*O:*9_ interacts not only with the glycan residues of the glycoprotein Vn but also with the carbohydrate polymer heparin. Interactions with glycans are employed by many pathogens for adhesion and invasion, especially in viruses (Marks et al., 2001; Guan et al., 2017; Sorin et al., 2021). Also, bacterial virulence factors like UpaB and Pili have been shown to interact with the glycosylations of glycoproteins and GAGs (Rajas et al., 2017; Paxman et al., 2019; Sauer et al., 2019; Vizarraga et al., 2021). To our knowledge, this is the first time that a trimeric autotransporter adhesin is described to bind glycans.

While it has been established in earlier work that YadA_*O:*9_ interacts directly with human Vn (Mühlenkamp et al., 2017), we show that recombinant Vn expressed in *E. coli* is not bound by YadA_*O:*9_. Eukaryotic proteins expressed in *E. coli* often lack glycosylations, as *E. coli* does not possess the glycosyltransferases and glycosidases present in eukaryotes (Sahdev et al., 2008; Khow and Suntrarachun, 2012). We further show in deglycosylation experiments that properly deglycosylated Vn was not bound by YadA_*O:*9_ any longer. This, and the fact that binding does not occur when using YadA_*O:*8_, further supports our model that the YadA_*O:*9_ loop is responsible for interactions with glycans. Furthermore, as Mühlenkamp et al. (2017) had described that heparin could inhibit the interaction between YadA_*O:*9_ and Vn, we set out to investigate whether heparin binding to Vn was actually the reason for this inhibition or whether a more direct interaction of heparin with YadA_*O:*9_ was the reason for this effect. While Vn indeed harbors a heparin binding site, this site is hidden in globular Vn (Izumi et al., 1989). We thus checked for binding of fluorescent *E. coli* expressing full-length YadA_*O:*9_ after preincubating either Vn with heparin-disaccharide or after preincubating fluorescent *E. coli* expressing YadA_*O:*9_ with heparin-disaccharide. Heparin preincubation of fluorescent *E. coli* expressing YadA_*O:*9_ inhibited Vn interaction, while preincubation of Vn with heparin-disaccharide did not. This is in agreement with literature stating that only 2% of the overall plasma Vn is present in a heparin-binding-competent state (Izumi et al., 1989), as well as with our model stating that the YadA_*O:*9_ 31-aa loop might be responsible for glycan binding. Furthermore, heparin-disaccharide was able to dissolve the YadA-mediated autoaggregation of bacteria expressing YadA_*O:*9_, which again indicates that there might be a direct interaction between heparin and YadA_*O:*9_. Finally, we were able to directly show the interaction using YadA_*O:*9_-expressing *E. coli* as well as purified YadA_*O:*9_ head domains in dot blots and ELISA-like assays. When looking at the YadA_*O:*9_ sequence, one can see that the loop contains seven positively charged residues (Arg and Lys). We hypothesize that the interaction between YadA_*O:*9_ could be explained by charge interactions with these residues, as the terminal sugar of the glycosylation of Vn is in most cases negatively charged sialic acid (Hwang et al., 2014). In heparin, sulfate moieties render this oligosaccharide heavily negatively charged (Rabenstein, 2002). It is worth noting that many known heparin binding motifs exhibit multiple, evenly spaced basic residues (Capila and Linhardt, 2002). Overall, we thus suggest that electrostatic interactions between the positively charged YadA_*O:*9_ loop residues and negatively charged functional groups on glycans are key to the binding affinity between YadA_*O:*9_ and glycans.

In terms of biological relevance, we hypothesize the interaction with YadA_*O:*9_ to be an additional mechanism for binding to host cell surfaces. As many secreted eukaryotic proteins are glycosylated for protein stability in the extracellular space (Varki et al., 2009), glycosylated ECM proteins could make a prime adhesion target during infection. Furthermore, a major group of molecules found in the ECM are GAGs such as heparin and heparan sulfate (Frantz et al., 2010). In addition to glycan adhesion being beneficial for the pathogen, it can conceptually be used in diagnostic workflows, e.g., to enrich pathogens from biological samples and potentially to develop anti-infective drugs. As glycans play a crucial role in pathogen adhesion, they have been used previously as therapeutics. Examples are the use of D-mannose in the treatment of urinary tract infections or of glycan derivatives to treat influenza (Domenici et al., 2016; Rustmeier et al., 2019; Weiss et al., 2020).

## Data Availability Statement

The original contributions presented in the study are included in the article/Supplementary Material, further inquiries can be directed to the corresponding author.

## Author Contributions

IM: data acquisition, data visualization, methodology, writing of the original draft, and project conceptualization. JL-B: data acquisition, methodology, data visualization, and draft writing and review. PM, MS, and SP: conceptualization, and draft review and editing. DL: project administration, project conceptualization, and draft writing and editing. All authors contributed to the article and approved the submitted version.

## Conflict of Interest

The authors declare that the research was conducted in the absence of any commercial or financial relationships that could be construed as a potential conflict of interest.

## Publisher’s Note

All claims expressed in this article are solely those of the authors and do not necessarily represent those of their affiliated organizations, or those of the publisher, the editors and the reviewers. Any product that may be evaluated in this article, or claim that may be made by its manufacturer, is not guaranteed or endorsed by the publisher.
