# Supplementary material for: The Trimeric Autotransporter Adhesin YadA of Yersinia enterocolitica Serotype O:9 Binds Glycan Moieties
Source: Front Microbiol. 2022 Feb 1;12:738818. doi: 10.3389/fmicb.2021.738818 (PMC8844515; doi:10.3389/fmicb.2021.738818)
Supplement: Supplementary file 1 [file Data_Sheet_1.PDF]

Supplementary Material

The trimeric autotransporter YadA of *Yersinia enterocolitica* serotype O:9 binds glycan moieties

1 Supplementary Figures

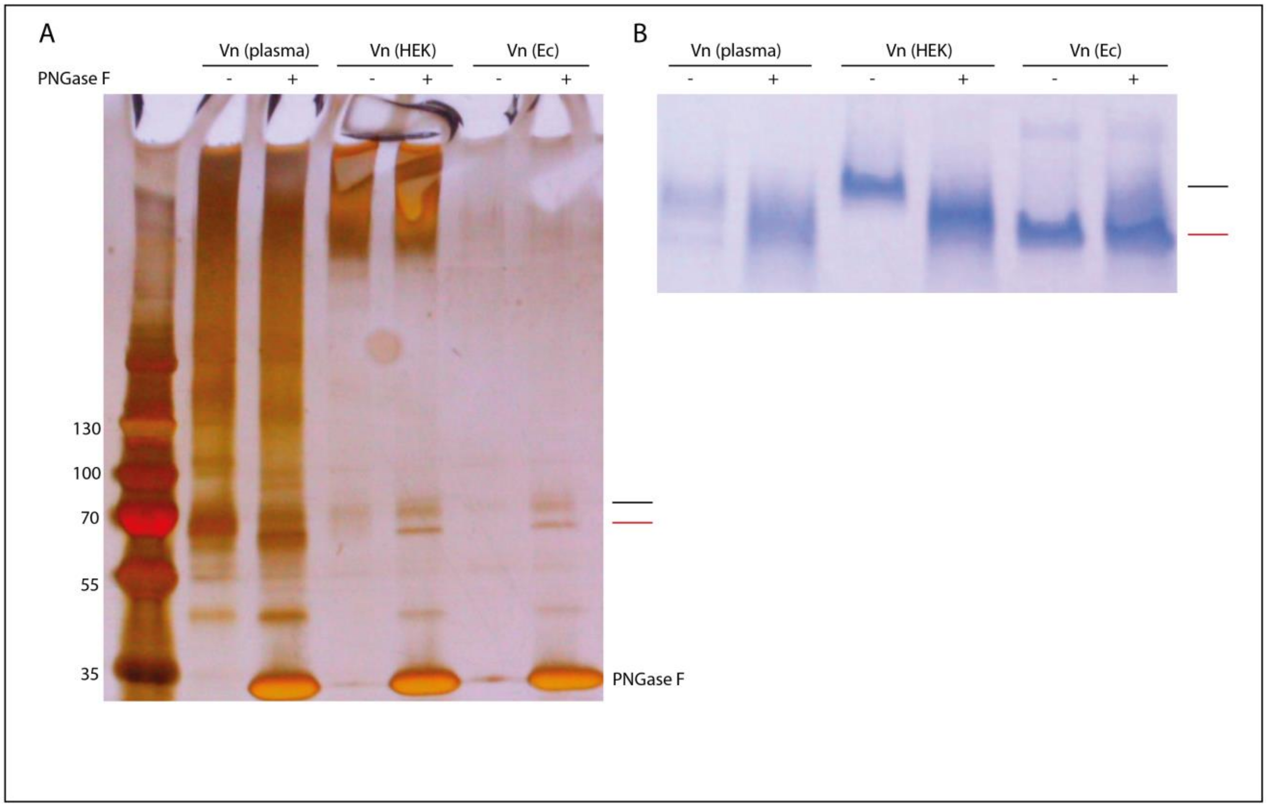

Supplementary Figure 1. *SDS PAGE gel showing deglycosylation of Vn.* **A)** Silver stained SDS PAGE gel of Vn samples before and after PNGase digest. The black line indicates glycosylated Vn, the red line indicates deglycosylated/non-glycosylated Vn. Folded Vn was digested but the samples were heated to 95 °C for 5 min before running on the SDS PAGE. Glycosylated samples do not fix well in the gel and are therefore not well visible. **B)** Coomassie stained SDS PAGE gel of Vn before and after PNGase digest. Here the Vn samples were unfolded by treatment with 1 % SDS and heated before the PNGase digest. The black line indicates glycosylated Vn, the red line indicates deglycosylated/non-glycosylated Vn. The glycosylated samples fix better in these gels. For the experiments shown in the manuscript, we deglycosylated folded Vn as shown in 1A.

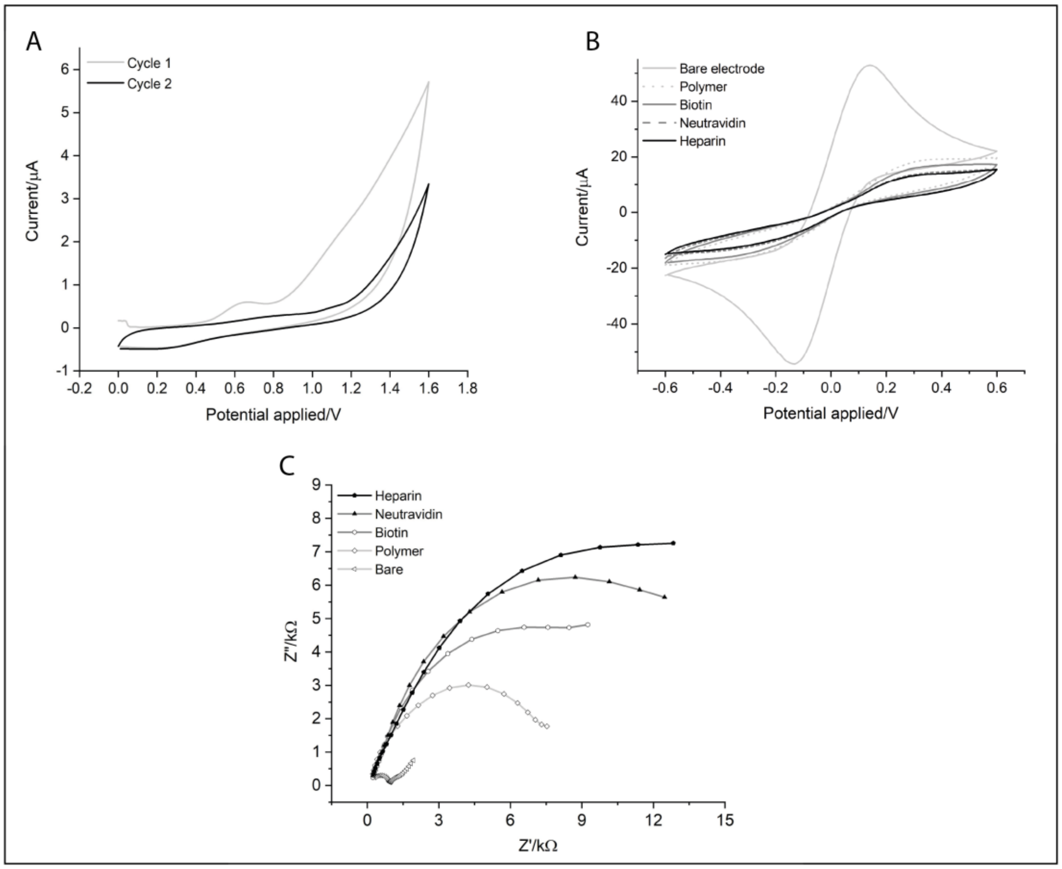

Supplementary Figure 2. *Characterization of EIS biosensor surfaces.* **A)** Electropolymerization of 2.5 mM octopamine in 10 mM Phosphate buffer pH 7.2. The electrode was cycled from 0.0 V to 1.6 V for two cycles at a scan rate of 100 mV/s. **B)** Cyclic voltammogram profile of biosensor layer construction: bare gold working electrode surface (solid light gray), POct (polymer) (dotted light gray), biotin (solid mid-gray), Neutravidin (dashed mid-gray) and heparin (solid black). The CV was cycled from E1 to E2 from -0.6 V to +0.6 at a scan rate of 100 mv/s in 10 mM $[Fe(CN)6]^{3-/4-}$ in 10 mM PBS, pH 7.2. **C)** Nyquist plot of layer-by-layer biosensor construction: bare gold working electrode surface (open tringle), POct (polymer) (open diamond), biotin (open pentagon), Neutravidin (solid triangle) and heparin (solid circle). All Nyquist profiles derived from EIS measurements in 10 mM [Fe(CN)6]3-/4- in 10 mM PBS, pH 7.2. EIS was recorded at 0 V over a frequency range of +0.1 Hz to +5 kHz, with a modulation voltage of +10 mV.

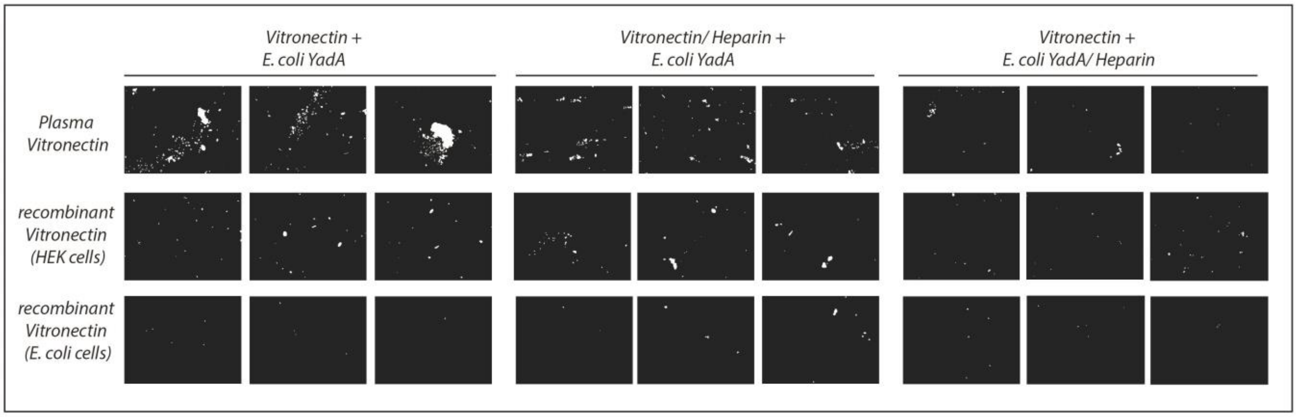

Supplementary Figure 3. *Representative binary images used for quantification of microscopy shown in Fig. 3.* Three representative, individual images for each quantification are shown. The micrographs were converted into binary images and quantified using Fiji. For plotting and statistical analysis the mean area ± SEM were plotted.

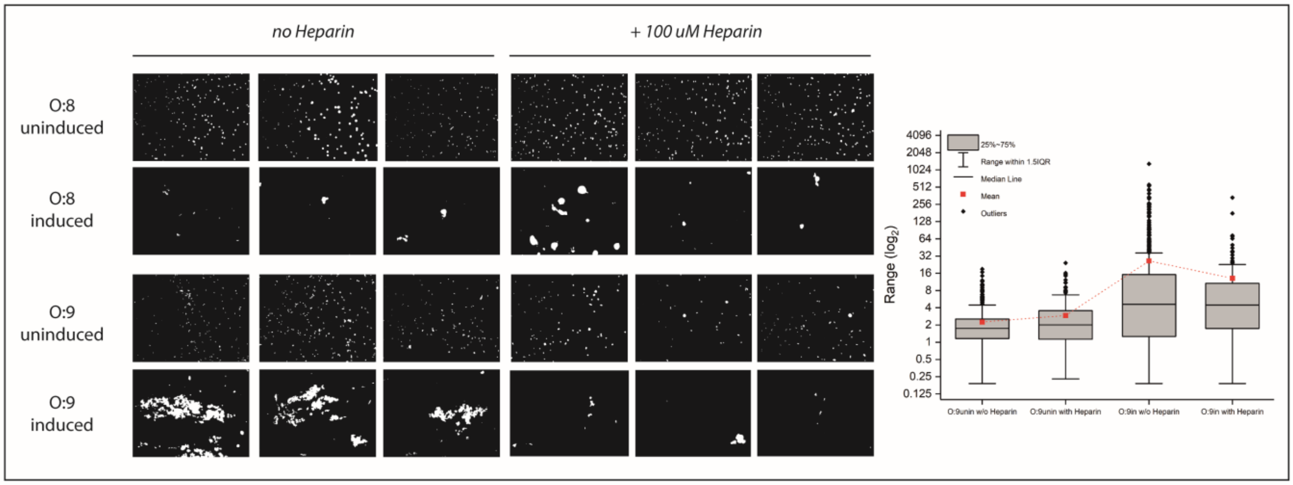

Supplementary Figure 5. *Representative binary images used for quantification of microscopy shown in Fig. 4.* Disaggregation of large auto-aggregates was quantified. Three representative, individual images for each quantification are shown. The image files were converted into binary files. The areas of the particles (auto-aggregates) were calculated using Fiji. The data are shown as Whiskers-plot with a log_2 scale. Samples of *E. coli* expressing $YadA_{O:8}$ with and without the addition of Heparin-disaccharide are shown in the two left Whiskers plots, samples of *E. coli* expressing $YadA_{O:9}$ with and without the addition of Heparin-disaccharide are shown in the two right whiskers plot.
